# A randomized controlled trial investigating effects of an individualized pedometer driven walking program on chronic low back pain

**DOI:** 10.1186/s12891-021-04060-8

**Published:** 2021-02-19

**Authors:** Angelica E. Lang, Paul A. Hendrick, Lynne Clay, Prosanta Mondal, Catherine M. Trask, Brenna Bath, Erika D. Penz, Samuel A. Stewart, G. David Baxter, Deidre A. Hurley, Suzanne M. McDonough, Stephan Milosavljevic

**Affiliations:** 1 College of Medicine, University of Saskatchewan, Saskatoon, Canada; 2 School of Health Sciences, University of Nottingham, Nottingham, UK; 3 School of Physiotherapy, University of Otago, Dunedin, New Zealand; 4 KTH Royal Institute of Technology, Stockholm, Sweden; 5 Faculty of Medicine, Dalhousie University, Halifax Regional Municipality, Nova Scotia Canada; 6 School of Public Health, Physiotherapy and Sports Science, University College Dublin, Dublin, Ireland; 7 RCSI University of Medicine and Health Sciences, Dublin, Ireland

**Keywords:** Chronic low back pain, Walking, Intervention, Physical therapy

## Abstract

**Background:**

Walking is an easily prescribed physical activity for people with low back pain (LBP). However, the evidence for its effectiveness to improve pain and disability levels for people with chronic low back pain (CLBP) within a community setting has not been evaluated. This study evaluates the effectiveness of a clinician guided, pedometer-driven, walking intervention for increasing physical activity and improving clinical outcomes compared to education and advice.

**Methods:**

Randomized controlled trial recruiting *N* = 174 adults with CLBP. Participants were randomly allocated into either a standardized care group (SG) or pedometer based walking group (WG) using minimization allocation with a 2:1 ratio to the WG. Prior to randomization all participants were given a standard package of education and advice regarding self-management and the benefits of staying active. Following randomization the WG undertook a physiotherapist guided pedometer-driven walking program for 12 weeks. This was individually tailored by weekly negotiation of daily step targets. Main outcome was the Oswestry Disability Index (ODI) recorded at baseline, 12 weeks, 6 and 12 months. Other outcomes included, numeric pain rating, International Physical Activity Questionnaire (IPAQ), Fear-Avoidance Beliefs Questionnaire (FABQ), Back Beliefs questionnaire (BBQ), Physical Activity Self-efficacy Scale, and EQ-5D-5L quality of life estimate.

**Results:**

*N* = 138 (79%) participants completed all outcome measures at 12 weeks reducing to *N* = 96 (55%) at 12 months. Both observed and intention to treat analysis did not show any statistically significant difference in ODI change score between the WG and the SG at all post-intervention time points. There were also no significant between group differences for change scores in all secondary outcome measures. Post hoc sensitivity analyses revealed moderately disabled participants (baseline ODI ≥ 21.0) demonstrated a greater reduction in mean ODI scores at 12 months in the WG compared to SG, while WG participants with a daily baseline step count < 7500 steps demonstrated a greater reduction in mean ODI scores at 12 weeks.

**Conclusions:**

Overall, we found no significant difference in change of levels of (ODI) disability between the SG and WG following the walking intervention. However, ODI responses to a walking program for those with moderate levels of baseline disability and those with low baseline step count offer a potential future focus for continued research into the benefit of walking as a management strategy for chronic LBP.

**Trial registration:**

United States National Institutes of Health Clinical Trails registry (http://ClinicalTrials.gov/) No. NCT02284958 (27/10/2014).

## Background

Chronic low back pain (CLBP), defined as back pain that is present for greater than 3 months [1], is a highly prevalent and costly musculoskeletal (MSK) disorder. In Canada, low back pain (LBP) is a health burden estimated to be $12 billion annually [2, 3]. Four out of every five Canadians will experience low back pain (LBP) at some point in their life, and CLBP will afflict at least one in five [4]. Some provinces such as Saskatchewan (SK) record life and point prevalence for LBP as high as 84 and 28% respectively [5]. These numbers highlight the need for effective interventions for LBP.

CLBP is associated with several physical and psychological comorbidities making management a complex problem for clinicians. Additional musculoskeletal or neuropathic disorders and/or an increased likelihood of depression, anxiety, and sleep disorders are frequent [6]. Obesity increases the risk of LBP, and is strongly associated with the need for treatment of CLBP [7]. Although there are many interventions and strategies described for the management of this disorder, effective treatment appears to be elusive [8–10]. Medication, exercise therapy, spinal manipulation, and cognitive behavior therapy are all examples of treatments with conflicting results for management of back pain [11, 12]. The majority of national and international guidelines regarding the clinical management of CLBP also recommend interventions focusing on exercise, remaining active, and patient education [13].

Many Canadians live a sedentary lifestyle often linked to modern work demands and transportation requirements [14]. A simple strategy for increasing physical activity is to increase the daily time and distance spent walking. Walking, a fundamental human activity, may have positive effects on chronic pain and self-reported disability [15], but its use as an interventio n for CLBP is not well studied [16–18]. There is strong evidence for pedometer-based walking interventions as a strategy for increasing physical activity and enhancing quality of life for individuals with MSK disorders [19], and a previous feasibility study of pedometer-driven walking intervention for CLBP has reported high levels of patient satisfaction with positive physical and clinical outcomes [18]. However successful adherence to a walking intervention likely requires attention to patient beliefs, self-efficacy, and setting of step count goals [20, 21]. While recent systematic reviews have identified low to moderate evidence for the use of walking to manage pain and disability in CLBP [22, 23], they also highlighted a need for further high quality research to more clearly determine the effectiveness of walking interventions for management of CLBP.

## Methods

The aim of this study was to evaluate a clinician guided, pedometer-driven, walking intervention for increasing and sustaining physical activity as a potential treatment for the management of CLBP. The study’s objectives were:

1. To determine perceived levels of disability and baseline levels of walking activity in people with CLBP.
2. To determine the uptake and adherence to a pedometer-driven walking programme for people with CLBP.
3. To test the effectiveness of a walking programme to improve outcomes for CLBP compared to standardised back care education package.

### Trial design

A community-based, single blinded randomized clinical trial recruiting adults with CLBP residing in the Canadian province of Saskatchewan. Recruitment took place from April 2015 to December 2016 with full 12 month follow-up completed by January 2018. All methods were carried out in accordance with relevant guidelines and regulations. Ethical approval was granted by the University of Saskatchewan Biomedical Ethics Board (#14–218) and the trial was registered with the United States National Institutes of Health Clinical Trails registry NCT02284958 (27/10/2014). Written informed consent was obtained from all participants.

Recruitment strategies involved posters, flyers, clinical and public notice boards, newspapers, and electronic bulletin boards to inform potential participants either receiving clinical care for this condition or those managing on their own. Potential participants were screened for inclusion eligibility in the following manner. Firstly by the research assistant, where ability to physically participate in a walking program was evaluated using the physical activity readiness questionnaire (PAR-Q+) [24]. Any participant answering yes to one or more questions on the PAR-Q+, was advised to consult with their primary healthcare provider to obtain physical clearance prior to confirming study participation. Secondly by a research physiotherapist, with 22 years of musculoskeletal clinical experience, who undertook a clinical history focused on participants meeting inclusion criteria and determining whether any exclusion criteria were present. If eligible, participants provided written informed consent and completed a 1 week (minimum of 5 days) pedometer trial in order to determine mean daily baseline step count prior to randomization. Although our initial protocol set an inclusion threshold of < 7500 mean daily steps [25] a number of potential participants, who had a greater recorded mean daily step count, were keen to participate in the study and thus a pragmatic decision was made to include them if they met all other inclusion criteria. Following this decision to remove the step count threshold, 53 participants included in the study had a recorded mean daily baseline step count of ≥7500 steps. All participant meetings with both the research assistant and research physiotherapist took place within the administrative and clinical evaluation facilities of the Canadian Centre for Health and Safety in Agriculture at the University of Saskatchewan.

### Inclusion criteria

Males and females aged 18 years or over, experiencing low back pain (i.e. between the 12th costal margin and gluteal fold with or without associated leg pain) persisting for a minimum of 3 months, and capable of participating in a walking program [25].

### Exclusion criteria

Spinal surgery in the past 12 months; evidence of nerve root, spinal cord, or cauda equina compression; severe spinal stenosis indicated by signs of neurogenic claudication; grade 3 to 4 spondylolisthesis; fibromyalgia, or systemic/inflammatory disorder; as well as any other current lower extremity musculoskeletal injury or contraindication to increasing physical activity (PA) levels. The latter included any medical condition limiting exercise tolerance or pregnancy [25].

Following successful completion of screening, all participants recorded baseline outcome measures (described below) and met individually with the research physiotherapist for education and advice regarding self-management and the benefits of staying active. The information was standardized using ‘The Back Book’ [26, 27] encouraging a graded return to normal activities, addressing the nature of LBP, correcting unhelpful beliefs, and emphasizing the need to use prophylactic pain control medication to allow activity [28, 29].

Following receipt of this standard package of education and advice, each participant was randomly allocated into either the standardized care group (SG) or the walking group (WG) using a minimization allocation with a 2:1 ratio to the treatment/control arms. Group allocation occurred by the participant selecting one sealed, plain, opaque envelope containing the computer generated randomized allocation from a container of identical envelopes that were prepared by a research administrator independent to recruitment and independent to the meetings with the physiotherapist. The sealed opaque envelopes were sequentially numbered and when an envelope was chosen this identifier was allocated to the participant clinical record used by the research physiotherapist and the research assistant. As self-management precluded double blinding, only the assessor for outcome measurements was blinded to group allocation. The structure and reporting of this trial was guided by the CONSORT statement for clinical trials [30].

### Intervention

Participants randomized to the SG having received the standard package of education and advice were then followed up at 12 weeks, 6 and 12 months to record outcome measures for comparison to baseline. As pedometer driven walking has been shown to be a safe and effective behavior change tool for facilitating increasing physical activity in inactive adults [31] participants in the WG were prescribed a personalized pedometer-driven walking program wearing a Yamax DigiWalker CW-701™ pedometer [30] for a minimum of five consecutive days per week during the 12-week intervention, similar to the B2A intervention ([17, 18]. This device demonstrates excellent accuracy (r = 0.98) and high reliability (ICC = .37–.99) when walking at different speeds and on varying surfaces [32, 33]. The research physiotherapist phoned each participant at a prearranged time, each week, to discuss progress, document mean daily step count (as recorded in diaries) for the previous week, and negotiate a new daily step target for the subsequent week. In this way the walking program was tailored on a week by week basis to the individual, with outcome measures also recorded at 12 weeks, 6 and 12 months for comparison to baseline measures for within and between group comparisons. In order to verify the consistency and fidelity of the walking intervention a member of the research team observed the delivery and manner of the research physiotherapist interacting with randomly selected participants at both baseline and at 8 weeks.

### Power

A power calculation identified the need to recruit 174 participants for 80% power to detect an 8-point mean difference (sd = 15.3) between groups in levels of disability, as recorded by change in the Oswestry Disability Index (ODI) following the 12 week intervention. As we anticipated a 15% drop-out at the end of the 12 week intervention our aim was to recruit 200 participants.

Outcome measures - previously described in detail in the protocol manuscript [25].

**Primary Outcome**

- Modified Oswestry Disability Questionnaire (ODQ) – scored out of 50 – and recorded as the Oswestry Disability Index (ODI) as a percentage [34]. Higher scores indicate higher levels of perceived disability.

**Secondary outcomes**

- Pain score (first question of ODQ)) where 0 indicates no pain and 5 is the worst pain imaginable.
- Short form of the International Physical Activity Questionnaire (IPAQ) asking participants about the time spent being physically active over the past 7 days [35].
- The Fear-Avoidance Beliefs Questionnaire (FABQ) and its sub-scales for work and physical activity (FABQW & FABQPA) [36]. Higher scores indicate higher fear avoidance beliefs.
- Back Beliefs questionnaire (BBQ) assessing a participant’s beliefs about various aspects of the future as a consequence of LBP [37].
- Physical Activity Self-efficacy Scale: determining level of confidence in a participant’s ability to perform a behavior necessary to achieve an expected outcome [38].
- EQ-5D-5L EuroQol health survey as a quality of life estimate [39].

**Adherence to the walking intervention**

- Mean steps per day for each week of the study (WG only). Participants were asked to record their total steps each day and report the daily values to the physiotherapist at the weekly phone call.

### Statistical analysis

Baseline anthropometric and outcome measures for both groups are descriptively reported and statistically compared. Mean and 95%CI for outcome measures at each time point for both groups are presented descriptively. Mean outcome change scores (with 95%CI) are presented for both within and between group statistical comparisons. Multi-variable post-hoc sensitivity analyses are also presented for change in ODI for self-reported moderately disabled (baseline ODI ≥21) participants; as well as for change in ODI in participants whose daily baseline step count was < 7500 steps. Student’s t-test and Wilcoxon non-parametric tests are used to compare normally and non-normally distributed continuous variables, respectively between groups. To compare categorical variables between groups, Chi-square or Fisher’s exact test have been used as appropriate. Mixed-effects models of repeated measures have been fitted to compare changes of primary and secondary outcomes within and between groups at each visit from baseline. In these models the intercepts indicate average group effect when predictors/independent variables are set to zero (i.e. average effect without the influence of any covariate/independent variable). Multiple imputation has been performed using PROC MI in SAS for missing data in the intention to treat analysis [40]. A *p*-value of less than 0.05 was considered as statistically significant (two-sided). All statistical analyses were done using SAS 9.4 (SAS Institute Inc., Cary, NC, USA).

## Results

### Participant recruitment

Following a 20 month recruitment strategy 241 people with CLBP expressed initial interest in taking part in the study. After screening, 174 participants (104 females and 70 males) met the inclusion criteria and formally consented to take part. At 12 weeks 21% (*n* = 37) of participants had withdrawn; at 6 months 34% (*n* = 60); and at 12 months 44% (*n* = 77).

**Fig. 1:**
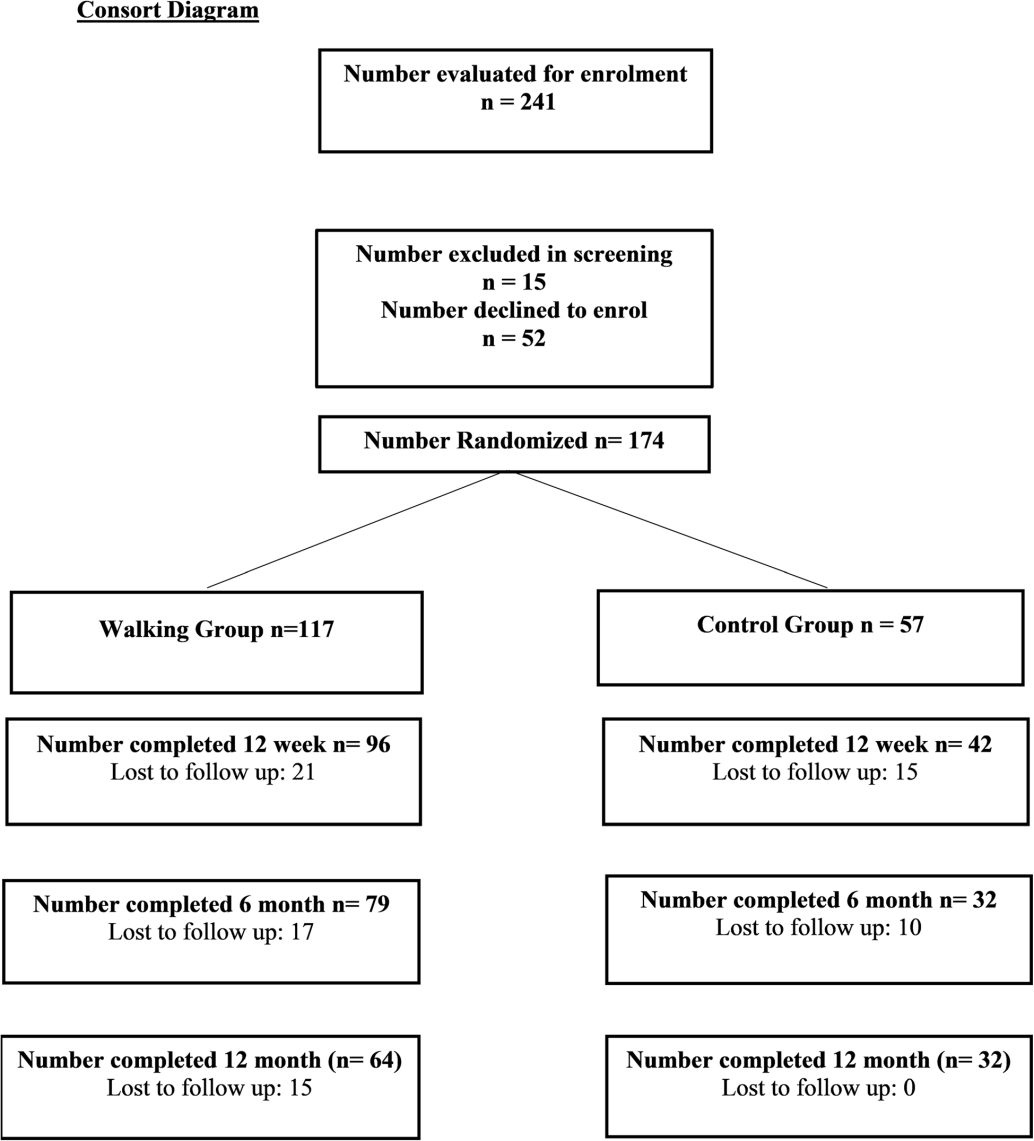
Consort Diagram

The Consort diagram (Fig. 1) demonstrates the flow of participants throughout the trial. Of the 52 individuals who declined to enroll, 29 did not attend their scheduled first or second appointment and could not be contacted for follow up. Other reasons for declining enrolment included time and family commitments, other health issues, sought treatment elsewhere, or not interested after reading the study information. For participants who did not complete the study, the majority were lost to follow up at one or more time points and did not provide a reason for withdrawal (*n* = 71). If the research assistant was able to contact the participant (13 out of the 71 participants who were lost to follow up; 8 from the WG, 5 from the SG), the most common reason reported for withdrawal was lack of satisfaction with the treatment, follow up procedure, or randomization to the control group. In the weekly phone calls between the WG participants and the research physiotherapist opportunity was made available for participants to report any adverse events from the walking intervention. At the completion of the 12 week intervention no adverse events had been recorded.

Table 1 provides baseline data for personal, anthropometric and outcome measures for all participants and presents a statistical comparison of these data for both WG and SG. The only statistically significant between group differences (*P* < 0.05) noted were for the EQ-5D-5L quality of life and the Physical Activity Self-efficacy Scale measures. Across all participants in the study mean baseline ODI score was 20.8 (SD 11.1) and mean baseline daily step count was 6193 (SD 2848) steps.

**Table 1:** Baseline comparison of WG to SG for personal, anthropometric and outcome measures

Variable (range)	All (***n*** = 174) *Mean (std)*	SG (***n*** = 57) *Mean (std)*	WG (***n*** = 117) *Mean (std)*	P=
**Age**	46.0 (16.5)	43.7 (17.2)	47.1 (16.1)	0.20
**BMI**	26.8 (5.9)	26.6 (5.9)	26.9 (5.8)	0.94
**BBQ** (9–45)	30.0 (6.7)	29.1 (6.0)	30.5 (7.0)	0.18
**EQ-5D-5L** (0.148–0.949)	0.788 (0.094)	0.798 (0.096)	0.769 (0.088)	0.03
**FABQ** (0–96)	24.8 (10.7)	24.8 (11.0)	24.7 (10.6)	0.96
**FABQ-PA** (0–24)	11.2 (5.5)	12.2 (5.9)	10.7 (5.2)	0.08
**FABQ-W** (0–42)	13.6 (8.7)	12.6 (9.3)	14.1 (8.4)	0.30
**IPAQ** (MET/min/week**)**	3046.0 (3910.9)	3461.6 (4623.1)	2836.3 (3501.5)	0.71
**ODI** (0–100)	20.8 (11.1)	21.2 (9.8)	20.5 (11.7)	0.69
**Pain** (0–5)	1.1 (0.8)	1.2 (0.7)	1.1 (0.9)	0.32
**Self-Efficacy (**1–25)	15.5 (3.9)	14.6 (3.3)	16.0 (4.1)	0.02
**Daily steps**	6193.2 (2848.0)	5976.6 (2783.1)	6291.2 (2883.3)	0.50
	N	N	N	
**Gender**				
F	104 (60.1%)	37 (64.9%)	67 (57.8%)	0.36
M	69 (39.9%)	20 (35.1%)	49 (42.2%)	
**ODI-Category**				
Missing	4		4	
< 21	95 (55.9%)	27 (47.4%)	68 (60.2%)	0.11
≥21	75 (44.1%)	30 (52.6%)	45 (39.8%)	
**Steps-Category**				
Missing	4	4		
< 7500	117 (68.8%)	36 (67.9%)	81 (69.2%)	0.86
≥7500	53 (31.2%)	17 (32.1%)	36 (30.8%)	

Key: *BMI* Body Mass Index (underweight to obese), *BBQ* Back Beliefs Questionnaire (higher scores = less fear), *EQ-5D-5L* Euroqol 5 dimension quality of life questionnaire (higher scores = higher perceived quality of life), *FABQ* Fear Avoidance Beliefs Questionnaire (higher score = more strongly held fear avoidance), *FABQ-PA* Fear Avoidance Beliefs Questionnaire (Physical Activity), *FABQ-W* Fear Avoidance Beliefs Questionnaire (Work), *IPAQ* International Physical Activity Questionnaire (higher score = higher estimated levels of physical activity), *ODI* Oswestry Disability Index (higher score = higher description of perceived disability), *Self-Efficacy Questionnaire* (higher scores = stronger self-efficacy beliefs)

Table 2 shows the mean scores (+/− 95%CI) for each outcome measure at each time point for both the SG and WG.

**Table 2:** Outcome measures – mean scores (−/+ 95%CI) baseline to 12 months

Outcome (range)	Group	Baseline (95%CI)	3 Month (95%CI)	6 Month (95%CI)	12 Month (95%CI)
**ODI (0 to 100)**	**WG**	20.5 (18.4 to 22.6)	15.1 (12.9 to 17.4)	13.1 (10.9 to 15.4)	11.9 (9.3 to 14.4)
	**SG**	21.2 (18.7 to 23.8)	18.8 (15.8 to 21.8)	16.8 (13.5 to 20.1)	16.7 (11.9 to 21.4)
**IPAQ (met-minutes/week)**	**WG**	2864 (2201 to 3470)	3212 (2645 to 3779)	2494 (1906 to 3082)	2658 (1844 to 3472)
	**SG**	3462 (2261 to 4662)	2322 (1484 to 3159)	1799 (1272 to 2327)	3016 (1671 to 4361)
**FABQ (0 to 96)**	**WG**	24.7 (22.8 to 26.6)	19.1 (16.7 to 21.4)	18.8 (15.7 to 21.9)	16.7 (13.7 to 19.6)
	**SG**	24.8 (22.0 to 27.7)	22.6 (19.3 to 26.0)	19.8 (15.8 to 23.8)	18.3 (14.4 to 22.1)
**FABQ (W) (0 to 42)**	**WG**	14.1 (12.5 to 15.6)	11.1 (9.3 to 12.9)	10.6 (8.5 to 12.8)	9.7 (7.7 to 11.8)
	**SG**	12.6 (10.2 to 15.0)	11.2 (8.4 to 13.9)	11.1 (7.8 to 14.4)	8.4 (5.4 to 11.5)
**FABQ (PA) (0 to 24)**	**WG**	10.7 (9.7 to 11.6)	8.0 (6.9 to 9.0)	8.0 (6.8 to 9.3)	6.9 (5.6 to 8.2)
	**SG**	12.2 (10.7 to 13.7)	11.7 (9.8 to 13.6)	8.7 (6.8 to 10.5)	9.8 (7.6 to 12.0)
**BBQ (9 to 45)**	**WG**	30.5 (29.2 to 31.8)	31.6 (30.2 to 33.1)	31.7 (29.8 to 33.7)	31.8 (30.1 to 33.6)
	**SG**	29.1 (27.5 to 30.6)	30.5 (28.7 to 32.3)	30.2 (27.3 to 33.1)	32.3 (29.8 to 34.8)
**ODQ Pain Rating (0 to 5)**	**WG**	1.1 (0.9 to 1.3)	1.0 (0.9 to 1.2)	0.9 (0.8 to 1.1)	0.9 (0.7 to 1.1)
	**SG**	1.2 (1.0 to 1.4)	1.4 (1.1 to 1.7)	1.2 (0.8 to 1.5)	1.3 (0.9 to 1.7)
**EQ-5D-5L (range − 0.148 to 0.949)**	**WG**	0.798 (0.781 to 0.815)	0.832 (0.762 to 0.811)	0.844 (0.823 to 0.864)	0.846 (0.823 to 0.868)
	**SG**	0.769 (0.746 to 0.793)	0.787 (0.762 to 0.811)	0.810 (0.779 to 0.841)	0.812 (0.773 to 0.852)
**Self Efficacy Scale (1 to 25)**	**WG**	16.0 (15.3 to 16.8)	15.1 (12.8 to 17.3)	15.2 (14.2 to 16.2)	15.9 (15.0 to 16.9)
	**SG**	14.6 (13.7 to 15.4)	15.0 (13.8 to 16.2)	13.5 (11.8 to 15.3)	14.9 (13.8 to 16.0)

**Fig. 2:**
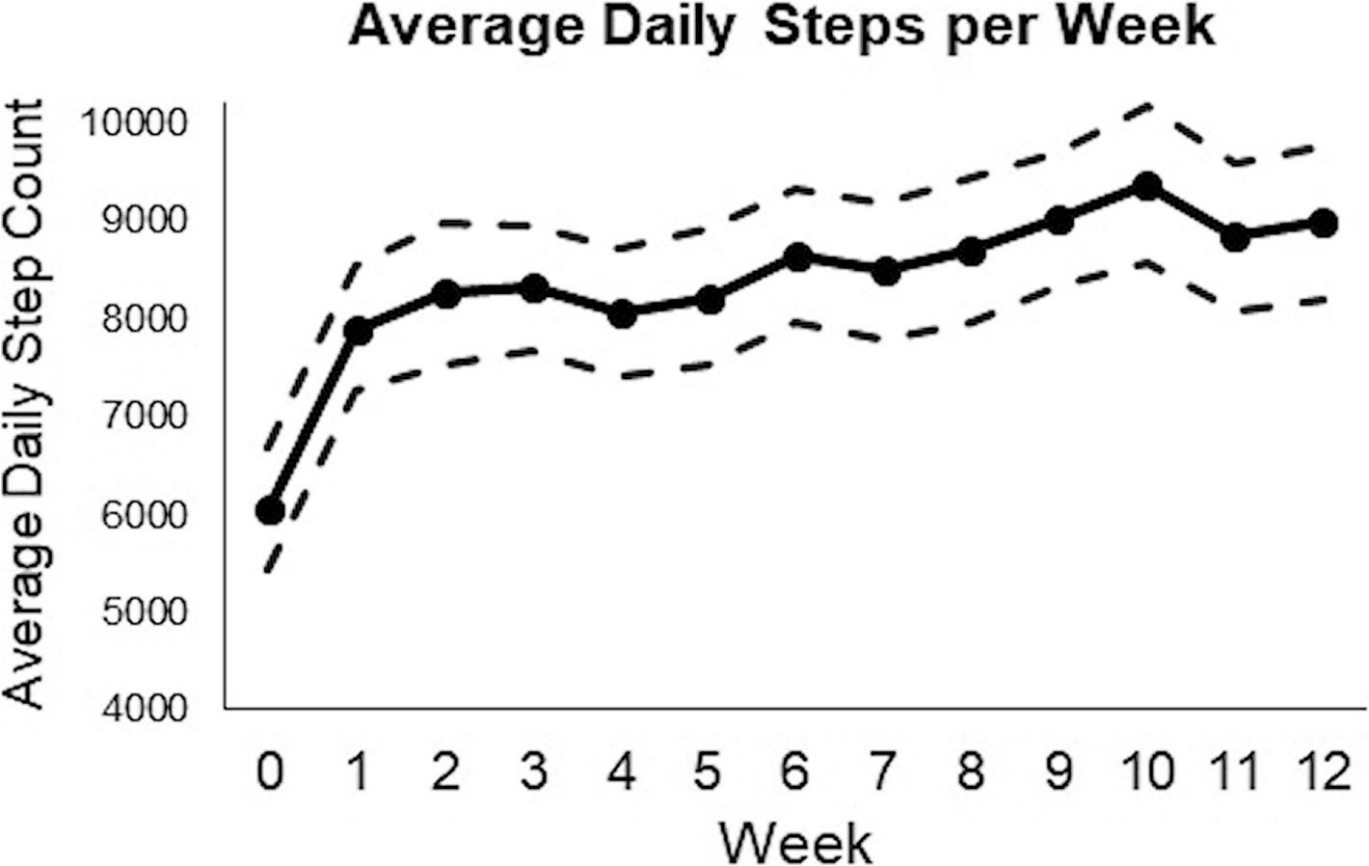
Average daily steps per week

From the perspective of adherence, participants in the WG significantly increased their daily step count over the 12 week period of the intervention by an average of 2140 (SD 2894) steps (34% increase from baseline). Change in group mean weekly step count (+/− 95%CI) is presented in Fig. 2 demonstrating a marked increase at Week 1 and a consistent but more gradual increase from week 1 to week 12.

Table 3 displays the results of the multivariable mixed effects model comparing the WG to the SG for change in all outcomes at all time points from baseline to 12 months. For the ODI primary outcome the results show that both groups improved significantly over the 12-week intervention. Greater changes in the ODI scores in favour of the WG at both 6 months (3.3) and 12 months (3.1) did not meet statistical significance.

**Table 3:** Multivariable mixed effects model comparing WG to SG for change in all outcomes from baseline to 12 months

	COVARIATES			CHANGE SCORE						
	Baseline	Estimate (−/+ 95%CI)	p=		3 month (−/+ 95%CI)	p=	6 month (−/+ 95%CI)	p=	12 month (−/+ 95%CI)	p=
**ODI**	**Intercept WG**	9.9 (2.8 to 17.0)		**WG**	−5.6 (−7.4 to −3.8)	< 0.0001	− 7.2 (−9.2 to − 5.2)	< 0.0001	−7.8 (− 8.9 to − 6.7)	< 0.0001
	**Intercept SG**	11.4 (4.1 to 18.7)	0.36	**SG**	−2.8 (− 5.5 to − 0.1)	0.04	− 3.9 (− 6.8 to − 1.0)	0.01	−4.7 (− 6.3 to − 3.1)	0.003
				**WG-SG**	−2.8 (− 6.1 to 0.5)	0.09	−3.3 (− 6.8 to 0.2)	0.06	− 3.1 (− 5.0 to − 1.2)	0.10
	**Gender(M)**	−1.7 (− 4.6 to 1.2)	0.23							
	**Age**	0.22 (0.14 to 0.30)	< 0.0001							
	**BMI**	0.03 (−0.21 to 0.27)	0.8							
**IPAQ**	**Intercept WG**	3676.7 (1718.1 to 5635.3)		**WG**	377.7 (− 396.9 to 1152.3)	0.34	−322.1 (− 1147.5 to 503.3)	0.44	− 136.9 (− 1003.2 to 729.4)	0.76
	**Intercept SG**	4311.9 (2305.3 to 6318.5)	0.23	**SG**	−843.7 (− 1989.7 to 302.3)	0.15	− 1386.0 (− 2565.7 to − 206.3)	0.22	− 202.2 (− 1516.0 to 1111.6)	0.76
				**WG-SG**	− 1222.3 (− 2605.9 to 161.3)	0.08	− 1063.9 (− 2503.1 to 375.3)	0.15	−65.3 (− 1640.7 to 1510.1)	0.94
	**Gender(M)**	1334.1 (552.1 to 2116.1)	0.0009							
	**Age**	−14.6 (−38.1 to 8.9)	0.22							
	**BMI**	−25.5 (−89.0 to 38.0)	0.43							
**FABQ**	**Intercept WG**	16.6 (9.0 to 24.2)		**WG**	−5.4 (−7.8 to −3.0)	< 0.0001	− 5.7 (−8.1 to − 3.3)	< 0.0001	−7.5 (−10.0 to − 5.0)	< 0.0001
	**Intercept SG**	17.2 (9.4 to 25.0)	0.75	**SG**	−1.6 (−4.9 to 1.7)	0.34	− 4.5 (− 8.0 to − 1.0)	0.01	− 5.4 (−9.1 to − 1.7)	0.005
				**WG-SG**	3.8 (−0.3 to 7.9)	0.07	1.2 (− 3.1 to 5.5)	0.59	2.1 (−2.6 to 6.8)	0.38
	**Gender(M)**	3.1 (0.0 to 6.2)	0.05							
	**Age**	0.06 (−0.04 to 0.16)	0.23							
	**BMI**	0.15 (−0.09 to 0.39)	0.24							
**FABQ (PA)**	**Intercept WG**	8.6 (5.1 to 12.1)		**WG**	−2.8 (−4.0 to −1.6)	< 0.0001	−2.8 (− 4.2 to − 1.4)	< 0.0001	− 2.3 (−3.7 to − 0.9)	< 0.0001
	**Intercept SG**	10.3 (6.8 to 13.8)	0.06	**SG**	−0.5 (− 2.3 to 1.3)	0.06	−3.1 (−5.1 to − 1.1)	0.001	−2.2 (− 4.2 to − 0.2)	0.02
				**WG-SG**	−2.3 (− 4.5 to − 0.1)	0.03	−0.3 (− 2.1 to 1.5)	0.80	0.1 (− 2.3 to 2.5)	0.30
	**Gender(M)**	1.0 (−0.4 to 2.4)	0.15							
	**Age**	0.02 (−0.01 to 0.05)	0.30							
	**BMI**	0.02 (−0.01 to 0.05)	0.72							
**FABQ(W)**	**Intercept WG**	8.1 (2.0 to 14.2)		**WG**	−2.6 (−4.2 to −1.0)	0.002	−3.0 (− 4.8 to − 1.2)	0.001	−4.0 (−6.0 to − 2.0)	< 0.0001
	**Intercept SG**	7.0 (0.9 to 13.1)	0.45	**SG**	−0.9 (−3.3 to 1.5)	0.48	−1.4 (−3.9 to 1.1)	0.29	−3.0 (−5.7 to − 0.3)	0.03
				**WG-SG**	1.8 (−1.1 to 4.7)	0.23	1.6 (−1.5 to 4.7)	0.31	1.0 (−2.3 to 4.3)	0.55
	**Gender(M)**	2.2 (−0.3 to 4.7)	0.08							
	**Age**	0.03 (−0.05 to 0.11)	0.42							
	**BMI**	0.13 (−0.07 to 0.33)	0.19							
**BBQ**	**Intercept WG**	32.2 (27.7 to 36.7)		**WG**	1.0 (−0.6 to 2.6)	0.19	1.3 (−0.3 to 2.9)	0.11	1.2 (−0.5 to 2.9)	0.16
	**Intercept SG**	30.7 (26.0 to 35.4)	0.20	**SG**	0.8 (−1.4 to 3.0)	0.49	0.4 (−2.0 to 2.8)	0.77	1.6 (−1.1 to 4.3)	0.23
				**WG-SG**	−0.2 (−2.9 to 2.5)	0.87	−1.0 (−3.9 to 1.9)	0.50	0.3 (−1.4 to 2.0)	0.16
	**Gender(M)**	−2.7 (−4.5 to −0.9)	0.005							
	**Age**	0.02 (−0.04 to 0.08)	0.40							
	**BMI**	−0.07 (− 0.23 to 0.09)	0.39							
**Self-Efficacy**	**Intercept WG**	16.1 (13.2 to 19.0)		**WG**	−1.1 (−1.9 to −0.3)	0.02	−1.3 (−2.3 to − 0.3)	0.008	− 0.4 (− 1.4 to 0.6)	0.40
	**Intercept SG**	14.7 (11.8 to 17.6)	0.04	**SG**	0.3 (−0.9 to 1.5)	0.69	−1.3 (−2.7 to 0.1)	0.07	−0.2 (− 1.6 to 1.2)	0.77
				**WG-SG**	1.3 (−0.3 to 2.9)	0.08	−0.02 (−1.8 to 1.4)	0.99	−0.4 (−2.2 to 1.4)	0.40
	**Gender(M)**	0.5 (−0.7 to 1.7)	0.36							
	**Age**	−0.0002 (− 0.0008 to 0.0004)	0.99							
	**BMI**	−0.01 (− 0.17 to 0.15)	0.81							
**EQ-5D-5L**	**Intercept WG**	0.832 (0.769 to 0.895)		**WG**	0.034 (0.016 to 0.052)	0.003	0.043 (0.023 to 0.063)	< 0.0001	0.038 (0.018 to 0.058)	0.0003
	**Intercept SG**	0.801 (0.772 to 0.830)	0.04	**SG**	0.020 (−0.007 to 0.047)	0.14	0.041 (0.012 to 0.070)	0.006	0.050 (0.021 to 0.079)	0.001
				**WG-SG**	0.013 (−0.018 to 0.044)	0.42	0.002 (−0.033 to 0.037)	0.91	0.012 (−0.023 to 0.047)	0.52
	**Gender(M)**	−0.006 (− 0.009 to − 0.004)	0.67							
	**Age**	−0.0007 (− 0.0014 to 0.0000)	0.09							
	**BMI**	−0.00002 (− 0.00004 to 0.0000)	0.98							

There were significant reductions in the FABQ and its two subunits for work and physical activity for both groups at each time point with a higher change score observed in the WG. However the between group differences did not meet statistical significance. Despite a higher change score improvement for the EQ-5D-5L quality of life measure noted for the WG there was no statistically significant between group differences.

Although there was an observable increase in IPAQ (377.7 mets/min) score above baseline measures for the WG at the 12 week time point the increase was not statistically significant. For the IPAQ, BBQ and Self Efficacy scale there were no significant within or between group changes from baseline at the 12 month time point.

Post hoc sensitivity analysis of participants whose baseline ODI ≥ 21 (classified as moderately disabled), demonstrated a statistically significant 13.6 (*p* < 0.0001) within group ODI reduction at 12 months for the WG and a 7.1 ODI difference compared to the SG (*p* = 0.01), however this between group effect was not observed at 12 weeks and 6 months. No other post hoc analyses were undertaken for ODI thresholds or for secondary outcome measures. A further post hoc sensitivity analysis for comparative between group changes in the ODI focused on participants with a mean daily step count of < 7500 steps. When using this cut point the WG demonstrated a statistically significant 5.9 (*p* < 0.0001) within group reduction and a 4.7 (*p* = 0.03) difference compared to the SG at completion of the 12 week intervention. While the magnitude of the reduction at 6 and 12 months also favoured the WG (7.1 v 3.1; and 8.3 v 3.4 respectively) the results were not statistically significant (*p* = 0.08 & *p* = 0.06).

## Discussion

This study tested the effectiveness of a walking programme to improve outcomes for people with CLBP compared to a pragmatic control group (i.e. a standardised back care education package). The results found no statistically significant difference in ODI scores at 12 weeks (completion of the intervention), 6 months (post baseline), and 1-year (post baseline) between the WG intervention and the SG groups. However, greater ODI change scores in favour of the WG at 12 weeks (2.8, *p* = 0.09), 6 months (3.3, *p* = 0.06) and 1-year (3.1, *p* = 0.08) were observed suggesting a clinician guided, pedometer based, walking intervention offers potential for clinical utility when compared to standardised advice to remain active.

The mean ODI score of 20.8 (SD 11.1) is considerably lower than other studies investigating disability levels of people with CLBP in the community where scores have ranged from 30.6 to 41.7 [18, 41–44]. Mean daily baseline step count 6193.2 (SD 2848.0) was also higher in the current study than values reported in a systematic review on pedometer interventions for CLBP which ranged from 2337 to 5563 steps [23] and is similar to the step count findings published by McDonough et.al [17].

From a population sample perspective we have also comparatively clustered (post-hoc) the participants in this study to the same 15 year age categories as described by Bath et al. in their exploration of national health survey data of ~ 25,000 Canadian adults aged 18 or older with CLBP [4]. Essentially, the percentage age distribution between the ages of 35 to 49 and 50 to 64 categories are the same (31 v 28% and 31 v 33% respectively). However, the current study proportionally has ~ 10% more participants in the 18 to 34 year cluster and ~ 10% less in the 65+ age category (27 v 17% and 12 v 22% respectively). BMI percentage distribution categories are similar for underweight/normal (39 v 40%), overweight (39 v 37%) and obese (22 v 23%) categories. The current study proportionally also has 8% more female participants (60 v 52%). Bath et al. do not have a record of ODI scores and data regarding lifestyle and health characteristics were also not recorded in the present study. Therefore, compared to prior research, the current study has a similar middle-aged distribution to the Canadian population with CLBP with a sample bias towards a younger distribution and a less representative sample for those aged 65+. This together with relatively lower levels of disability and higher daily step levels at baseline, as well as a higher percentage of females in the current study, may be important confounding factors that might be contributors to the non-significant ODI differences between the two groups. It is important to note that a recent review by Tudor-Locke et al. describes much lower baseline step counts (in disabled and elderly populations) and also recommends potential goal setting strategies for such people including those with musculoskeletal disorders [45]. Such observations and the findings of others are suggestive of potential future research directions for exploring walking based interventions for moderately disabled and low step count CLBP participants.

Further analyses of secondary outcome measures found no significant difference between groups at all-time points for the IPAQ, FABQ, FABQ(W), BBQ, Self-Efficacy Scale, and EQ-5D-5L. The only significant difference found was for FABQ (PA) at the 12 week point whereby the walking group demonstrated a greater significant decrease (− 2.3) compared to standardised education group (*p* = 0.03). A recent systematic review on the effects of walking in people with CLBP identified only two studies that assessed short-term fear avoidance and found the overall effect size of walking was very small and not significant compared to other exercise interventions [23]. These results highlight the need for further research into the psychological effects of walking in CLBP patients.

The current study also evaluated the uptake and adherence to a pedometer-driven walking programme for people with CLBP. One hundred and seventeen participants were allocated to the WG with 18% dropping out over the 12 week intervention and 54% completing outcomes at the 1 year point. The relatively low dropout rate for the WG (82% of participants completed the walking programme and 81% completed primary and secondary outcome measures at this 12 week time point) and the consistent weekly step count increase (Fig. 2), demonstrates that the intervention strategies used to encourage adherence during the intervention period were effective [25]; however, further research should investigate reasons for dropping out and identify potential mechanisms to improve longer term adherence. Furthermore, the mean 34% improvement in step count (2140 steps) for the WG over 12 weeks shows program delivery was effective as a method to change physical activity behavior, and comparable to the changes seen by McDonough et al. [18] which helped inform this intervention, and the mean increase of 1950 steps per day (range 818 to 2829 steps) reported in a review investigating the effects of pedometer-driven walking program on musculoskeletal disorders [19]. Interestingly, the SWIFT trial of supervised walking for managing CLBP [15] showed higher levels of adherence for a physiotherapy supported, but physiotherapy facilitated, walking programme compared to group based physiotherapy led exercise classes or individualised standard physiotherapy care.

The within group change score for the WG intervention demonstrated an ODI change score of 5.7 at 12 weeks and 7.8 at 1-year. The minimal clinically important differences (MCID) for the ODI reported in the literature ranges from a 50% change [43], a 30% change [46], a 10-point change [47] to as small as a 5-point change [48]. Post-hoc analyses on WG participants dichotomised [49] into low perceived disability (ODI < 21) at baseline (*n* = 95) and those who were (at least) moderately disabled at baseline (*n* = 75) (ODI ≥ 21) found a mean non-significant between group ODI difference at 12 weeks of 4.1 (*p* = 0.11) and a significant difference of 7.1 (*p* = 0.01) at 1-year for the ODI ≥ 21 sub-group. The effect of the walking intervention on change in ODI also appears to be comparatively stronger (4.7, *p* = 0.03) immediately following the 12 week intervention for participants (*N* = 117) whose mean daily baseline step count was less than 7500 steps while paradoxically, the influence of this step count threshold (while observable) was non-significant at 6 and 12 months. The post-hoc results demonstrating significant effects at 12 months in participants moderately disabled with CLBP, or alternatively at 12 weeks for WG participants with a baseline daily step count of < 7500 steps, although non-confirmative, offer potential for future research seeking to replicate these findings, whereby different subgroups of CLBP patients, in terms of level of perceived disability and/or mean daily step count, may respond differently to a community walking programme and could be investigated in a larger study with a more focused design.

A further scan of our data at the completion of the 12 week intervention demonstrate that proportionally more of the WG (25%, *N* = 29) achieved a ≥ 10% reduction in ODI compared to the SG (19%, *N* = 11). Proportionally more of the WG (25%, *N* = 29) also achieved a ≥ 2 point reduction in (ODI adjusted) pain scores compared to the SG (12%, *N* = 7). Hurley et al. report 45% of WG participants demonstrate a ≥ 10% points reduction in ODI following a 12-week intervention [15], and 39% achieved a ≥ 2 points NRS at 12 months. Other walking based studies report similar findings to the present study including an intervention advising patients to walk daily for 30 min reporting 26% (*n* = 9/35) of participants achieved ≥10% points reduction in ODI and 34% reduction (*n* = 12/35) on a pain rating visual analogue scale [50]. A supervised Nordic walking program also resulted in more modest changes in clinical outcomes with 17.5% (*n* = 7/40) of patients achieving an MCID for disability, and 25% (*n* = 10/40) for pain [51]. Overall, the results of the current research support findings from a systematic review which found low-to moderate-quality evidence that a walking intervention was as effective in terms of both pain and disability reduction when compared to other conservative treatments including other forms of exercise and education for patients with chronic LBP [22]. Importantly, the authors note that few studies included objective measures of (baseline and outcome) walking activity or undertook a sensitivity analysis of results to explore the effects of baseline activity and or disability levels on outcomes.

Our results indicate the potential for a physiotherapist guided and mentored daily walking program aimed at increasing mean daily step count to demonstrate positive clinical effects within chronic LBP patients with perceived (ODI rated) moderate levels of disability. The intervention is relatively simple and would require little specific training for physiotherapists and potentially involve screening of patients as well as resource allocation to allow effective follow-up of patients within a clinical environment. Future studies could include a focus on screening and targeting walking interventions in those patients with moderate disability and lower daily step counts to validate the findings of the current study.

### Strengths and limitations

We do not know if our community based recruitment strategy influenced CLBP participant motivation differently than if they were recruited via a clinical route, and may need to be considered when tailoring future walking interventions within a clinical population. However, the prevalence of CLBP in any given community is likely to be substantial, yet not all who report the condition will be seeking management for the disorder. There will likely be a number of reasons why treatment from a health professional is not being sought, including: self-management strategies, symptom tolerance, lack of previous treatment benefit, financial pressures, other accessibility barriers and a raft of other psychosocial factors [52–55]. We did not record who was currently receiving clinical management from a health professional or identify those who were self-managing their condition, and thus cannot identify whether this was a factor in the recruitment and retention rates in the study or their outcomes. We also did not undertake a comparative evaluation of step count for the SG at 12 weeks and thus cannot determine whether a standardized package of care can also influence mean daily step count over this period of time. Thus there is the potential for a positive bias response for the WG and a negative bias for the SG. At the completion of the study the research physiotherapist commented on how several SG participants voiced disappointment at not being included in the WG intervention. While the outcome measures used in this study were considered extensive, providing a reasonable profile of psychosocial parameters, a number of other measures such as pain duration, depression, sleep deprivation, work participation, level of education, current use of medication and level of education were not gathered or included. We were also unable to gather sufficient data from our participants relative to the costs of managing their disorder at 12 week, 6 and 12 month time points and were unable to undertake a meaningful cost comparison for between group effects.

We consider the powered sample size (*n* = 174) a strength of the study with an acceptable number of drop outs. This has allowed analyses with mixed effect models for both observed and imputed (intention to treat) data as well as allowing post-hoc analyses in order to help identify potential scenarios where the intervention may have most utility or most effect. Although there is potential for a type II error in accepting the null hypothesis at 12 weeks, where 37 participants had withdrawn from the study, an intention to treat analysis also found no significant difference between both groups at this time point, or at 6, or 12 months. Our study design also used only one physiotherapist to deliver the intervention, thus eliminating between-therapist variations in program delivery impacting the outcomes of the study. While the results of this study indicate promising potential for a community based walking intervention future research with an adequately powered sample size, focusing on moderately disabled ODI inclusion criteria and low daily step count [45], will be required. Design of such a study will also need to consider strategies to optimize recruitment and retention. Future trials should also consider investigating for differences or non inferiority/equivalence in clinical outcomes between a community walking interventions for patients with CLBP compared with traditional face-face treatment. Our results indicate the potential for positive benefits of a community walking programme and, considering the mode of delivery; may provide a relatively simple and clinically effective way to manage patients with CLPB.

## Conclusions

Overall, we observed no significant differences in pain and disability scores between the WG and SG groups at all time points of the study. The WG showed a significantly improved overall step count at 12 weeks compared to baseline, and those reporting moderate levels of disability with a low daily step count appeared to be more responsive to the walking intervention. This is the first study to identify different disability defined responses to a walking program in people with CLBP. Further mixed methods study is warranted to investigate baseline step counts employing objective measures of physical activity and levels of disability as key response variables to walking programs in people with CLBP in the community and determine what personal factors indicate individuals more likely to adhere to the program and have a successful outcome.

## Data Availability

The datasets generated and/or analysed during the current study are available from the Federated Research Data Repository at 10.20383/101.0296

## Abbreviations

LBP: Low Back Pain
CLBP: Chronic Low Back Pain
WG: Walking Group
SG: Standardized Care Group
ODQ: Oswestry Disability Questionnaire
ODI: Oswestry Disability Index
IPAQ: International Physical Activity Questionnaire
FABQ: Fear Avoidance Beliefs Questionnaire
FABQ(W): Fear Avoidance Beliefs Questionnaire (Work)
FABQ (PA): Fear Avoidance Beliefs Questionnaire (Physical Activity)
BBQ: Back Beliefs Questionnaire
EQ-5D-5L: Euroqol – 5 Descriptors – 5 Levels (quality of life measure)
MSK: Musculo-Skeletal
PAR-Q: Physical Activity Readiness Questionnaire
B2A: Back to Activity
NRS: Nominal Rating Scale -
